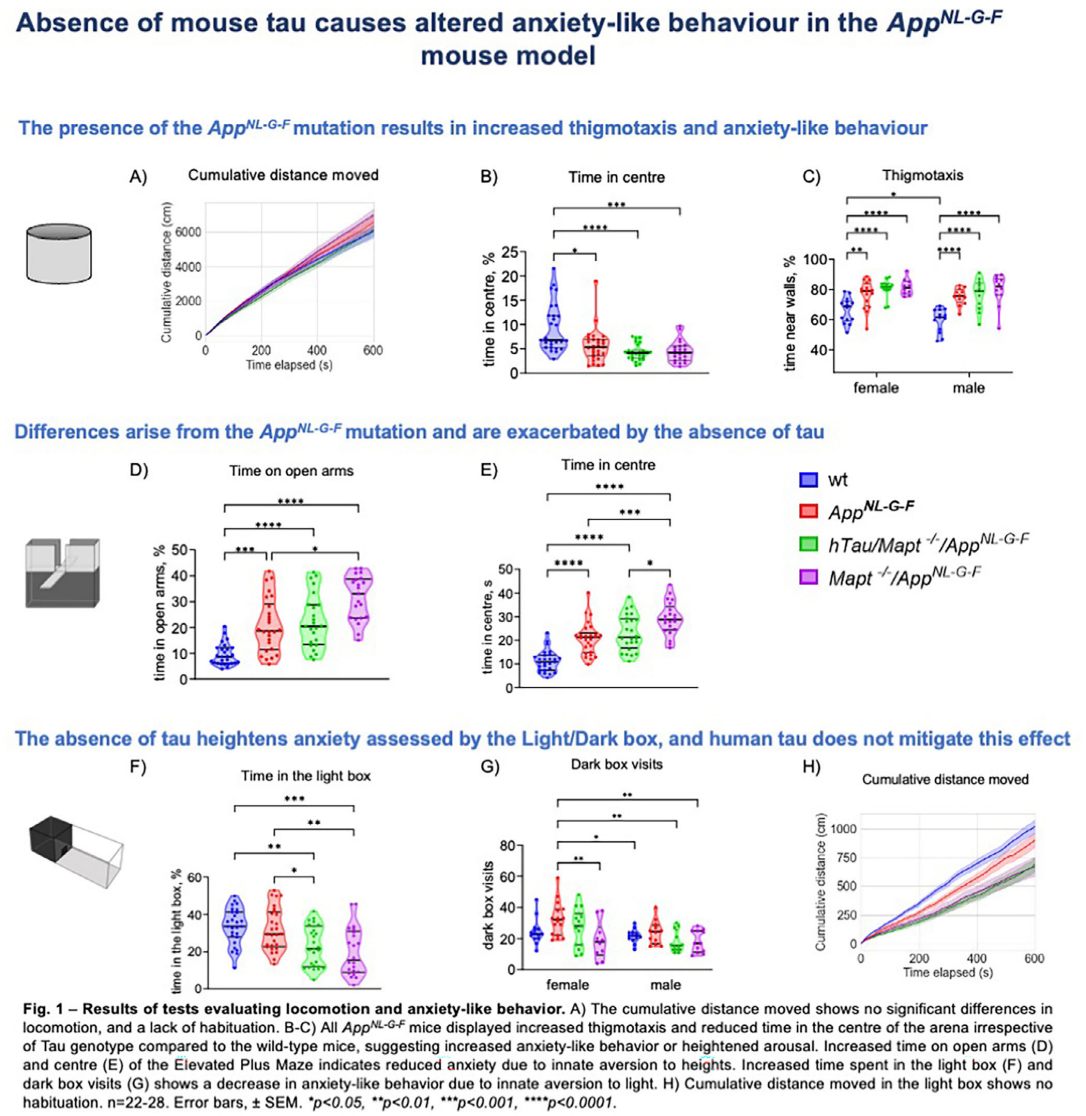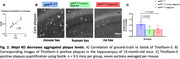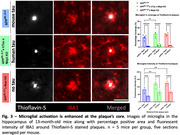# Tau absence in *App* ^NL‐G‐F^ mice alters amyloid aggregation and microgliosis without exacerbating memory loss

**DOI:** 10.1002/alz70861_108338

**Published:** 2025-12-23

**Authors:** Loukia Katsouri, Angela Misak, Georgia Ppasia, Diego Caron, Stephen Burton, John O'Keefe

**Affiliations:** ^1^ University College London, London, Greater London UK

## Abstract

**Background:**

Accurate models of Alzheimer’s disease are crucial for understanding disease mechanisms and therapeutic development. The overexpression of APP C‐terminal fragments in many transgenic mouse models introduces neuropathological artefacts, raising questions as to the interpretation of the relationship between amyloid‐beta (Aβ), tau and neuroinflammation. To better understand tau pathology alongside plaque deposition, we generated *hTau/Mapt^‐/‐^/App* ^NL‐G‐^ *^F^* mice which carry three familial *App* mutations and all six isoforms of human tau without mouse tau. Unlike other mouse models, *hTau/Mapt^‐/‐^/App* ^NL‐G‐F^ do not express FTD mutations nor overexpress APP, making this model specifically suited for studying the tauopathy in Alzheimer’s disease without effects of FTD pathology.

**Method:**

At twelve months of age, wild‐type, *App* ^NL‐G‐F^ *, hTau/Mapt^‐/‐^/App* ^NL‐G‐F^, and *Mapt^‐/‐^/App* ^NL‐G‐^ *^F^* mice underwent a comprehensive battery of behavioural tests, which yielded extensive data on locomotion, anxiety‐like behaviours, and memory. Machine‐learning techniques were employed to extract and analyse behavioural patterns. At the end of the experiments, we collected the brains and performed histopathological analyses. To explore the association between Aβ and Tau, core plaques and total Aβ were imaged using fluorescent microscopy. We conducted a quantitative, machine‐learning‐based image analysis employing Ilastik software and a Python script to quantify total Aβ and Thioflavin‐S‐positive plaques in the hippocampus and cortex. Furthermore, we developed an algorithm to analyse microglial coverage relative to plaque distance as well as astrocytic coverage at the plaque region in an unbiased quantitative way.

**Result:**

*App* ^NL‐G‐F^ exhibited impaired working memory, deficits in short‐term spatial memory, but normal locomotion and recognition memory. Loss of mouse tau, or the added presence of human Tau did not worsen memory. In addition, anxiety‐like behaviour was altered in *Mapt^‐/‐^/App* ^NL‐G‐F^ mice. This was not mitigated by the presence of human Tau (Figure 1). Absence of mouse tau or human tau overexpression did not affect total Aβ but significantly reduced Thioflavin‐S positive plaque load(Figure 2). Astrogliosis was unchanged, but we found altered microglial clustering differentially associated with diverse plaque morphology and proximity to plaques (Figure 3).

**Conclusion:**

There is interaction between tau and amyloid pathologies, with certain behavioural alterations linked to a tau‐independent amyloid pathology. The histological analysis suggests that the absence of mouse tau affects amyloid deposition and microgliosis.